# Study of rs12532, rs8670 Polymorphism of Msh Homeobox 1 (MSX1), rs61754301, rs4904155 Polymorphism of Paired Box Gene 9 (PAX9), and rs2240308 Polymorphism of Axis Inhibitor Protein 2 (AXIN2) Genes in Nonsyndromic Hypodontia

**DOI:** 10.1155/2019/2183720

**Published:** 2019-11-05

**Authors:** Krisztina Mártha, Bernadette Kerekes Máthé, Valeriu George Moldovan, Claudia Bănescu

**Affiliations:** ^1^Department of Orthodontics, George Emil Palade University of Medicine, Pharmacy, Science and Technology of Targu Mures, 38 Gh. Marinescu St, 540139 Târgu Mureș, Romania; ^2^Department of Tooth and Dental Arch Morphology, George Emil Palade University of Medicine, Pharmacy, Science and Technology of Targu Mures, 38 Gh. Marinescu St., 540139 Târgu Mureș, Romania; ^3^Genetics Laboratory, Center for Advanced Medical and Pharmaceutical Research, George Emil Palade University of Medicine, Pharmacy, Science and Technology of Târgu Mureș, 38 Gh. Marinescu St., 540139 Târgu Mureș, Romania

## Abstract

The etiology of hypodontia is complex, in which both genetic and environmental factors can be related. The main objective of our study was to contribute to elucidating the genetic background of nonsyndromic hypodontia (NSH). In this order, we selected 97 NSH subjects (70 females and 27 males) from patients referred to orthodontic treatment, and we matched to each NSH subject a control by age and sex. DNA was obtained from epithelial cells from the oral mucosa. Genotyping of the PAX9 (rs4904155 and rs61754301), MSX1 (rs8670 and rs12532), and AXIN2 (rs2240308) SNPs was performed by using TaqMan SNP Genotyping Assays on a real-time PCR system. Single-nucleotide polymorphisms (SNPs) were studied for the whole NSH group and for frontal and lateral agenesis NSH subjects separately. Our results showed that the variant genotype (*p*=0.0008, OR = 2.9, 95% CI = 1.58–5.3) and variant T allele (*p*=0.0002, OR = 2.65, 95% CI = 1.6–4.39) of the MSX1 rs8670 SNP increased the risk of hypodontia in the studied population when the whole NSH group was compared with controls. The variant genotype of the MSX1 rs8670 SNP was the most frequent in frontal agenesis; meanwhile in the lateral agenesis NSH group, the AXIN2 rs2240308 SNP showed a higher frequency of the variant genotype, with a trend towards statistical significance. In conclusion, the results of the present study showed that the variant genotype and variant T allele of the MSX1 rs8670 SNP increased the risk of hypodontia in the studied population. The presence of the variant A allele of AXIN2 rs2240308 is associated with frontal agenesis but not with lateral agenesis.

## 1. Introduction

Hypodontia or tooth agenesis is the most common developmental dental disorder, and it may consider the failure of the development of 1–6 teeth. The nonsyndromic hypodontia (NSH) is not associated with various genetic diseases [1]. Even in moderate forms of NSH, aesthetic and functional problems might be major, their treatment being considered an interdisciplinary challenge, and early diagnosis is the basis for prevention or reduction of complications [2].

The frequency of NSH (excluding third molar agenesis) shows values between 2.2 and 10.1% in Caucasian studies [3], and statistical differences are due to the provenience of subjects from different geographic areas, examination methods [4, 5], or selected subjects, NSH being more common among patients undergoing orthodontic treatment.

Odontogenesis is a complex process which involves many genes and signalling pathways; complex interaction of the genetic information with the environmental factors has been described. The development of teeth begins with an epithelial-mesenchymal interaction, each stage of which is genetically influenced. With more than 200 genes and nearly 300 molecules being involved in the odontogenetic process, at present, bone morphogenetic protein (BMP), fibroblast growth factors (FGF), and sonic hedgehog (SHH) and wingless integrated (WNT) ligand signalling pathways are recognized as the most important mediators of the epithelial-mesenchymal odontogenetic interaction, in the initiation, morphogenesis, and differentiation stages of tooth development [6, 7]. Most often reported genes associated with the familial form of the nonsyndromic tooth agenesis are paired box gene 9 (PAX9), Msh homeobox 1 (MSX1), ectodysplasin A (EDA), and axis inhibitor protein 2 (AXIN2) [8]. Probably because of the action of some physical, chemical, and biological factors on the genome, the single-nucleotide polymorphisms (SNPs) show variations between the studied groups.

Whilst it has been established that hypodontia is a genetically and phenotypically heterogeneous condition, advancements in molecular genetics allow us to identify the natural variations in the nucleotide sequence in all the genes that are involved in the odontogenetic process.

For MSX 1 gene mutations, the latest summary of reported SNPs reported 20 mutations, most of them being associated with autosomal dominant, nonsyndromic oligodontia [9]. Up to now, more than 50 mutations have been reported in the PAX9 gene, but no correlations between the genotype and the phenotype have been identified [10, 11].

One of the latest reviews concerning syndromic vs nonsyndromic tooth agenesis mutations identifies nine mutations in nonsyndromic tooth agenesis (TA), while 12 mutations were identified in syndromic TA and other conditions [12].

The present large-sample-size study aimed to investigate the prevalence, phenotype, and clinical forms of NSH and to analyse the association between PAX9 (rs4904155 and rs61754301), MSX1 (rs8670 and rs12532), and AXIN2 (rs2240308) SNPs and NSH, and with frontal and lateral agenesis in a European population.

## 2. Materials and Methods

### 2.1. Subject Selection and Sampling

After the approval of the Ethics Committee of the Scientific Research of our university (Approval no. 60/07.03.2018), two groups of subjects were selected: the NSH group and the control group (C), from patients referred to orthodontic treatment to the Department of Orthodontics of our university, namely, University of Medicine, Pharmacy, Science and Technology of Târgu Mureș, Romania, between April 2017 and March 2019.

All the 97 subjects from the NSH group were unrelated, healthy individuals with agenesis of one to five teeth, and for each NSH subject, a control matched by sex and age was included in the C group (that includes 97 individuals).

Complete and complex diagnosis of NSH was performed after detailed clinical and radiological examination; third molar agenesis cases were excluded.

NSH subject selection was made by well-defined inclusion criteria, and agenesis subjects were selected from patients referred to orthodontic treatment. Details regarding the two studied groups are presented in Table 1.

Prior to clinical, radiological, and genetic examination, informed consent was obtained from each subject, and oral swabs were coded to maintain confidentiality.

Epithelial cells from the oral mucosa were obtained using ethylene oxide-treated buccal swabs (Isohelix DNA Buccal Swabs, Isohelix Ltd., Kent, UK), and each tube that contained the oral swab was coded to maintain confidentiality and transported for DNA isolation and genotyping to the genetic laboratory.

### 2.2. Genotyping

Each DNA obtained from epithelial cells from the oral mucosa was quantified by the aid of the Eppendorf BioSpectrometer basic system and stored at −20 degrees until the genetic analysis. Genotyping of the PAX9 (rs4904155 and rs61754301), MSX1 (rs8670 and rs12532), and AXIN2 (rs2240308) SNPs was performed by using the corresponding predesigned TaqMan SNP Genotyping Assays on the 7500 Fast Dx Real-Time PCR Instrument.

### 2.3. Statistical Analysis

Data collection was done in Excel, and statistical analysis was performed using GraphPad InStat 3.1 (GraphPad Software, San Diego). Chi-square tests with Yates' correction were used for comparison of the studied genotypes between the NSH and C groups separately for all the subjects, for frontal and for lateral agenesis. Odds ratio (OR) was calculated to quantify the strength of the studied genotypes (*p* ≤ 0.001, highly significant; 0.001 ≤ *p* ≤ 0.05, significant; *p* ≥ 0.05, not significant). Confidence level (CI) was established at 95% (Woolf approximation was used).

## 3. Results

The female : male ratio was 1 : 2.59 in the NSH group and also in the control group because controls were selected to match by age and sex each NSH case.

Table 2 summarizes the genotype distributions and allele frequencies for the five SNPs we studied, comparing the NSH group with the control subjects. Significant differences in the distribution of MSX1 rs8670 SNP genotypes in NSH cases and controls were detected.

A significant association was observed between the heterozygous and homozygous variant genotype (C/T + T/T) of the MSX1 rs8670 SNP and the risk of developing NSH (*p*=0.0008, OR = 2.9, 95% CI = 1.58–5.3). No association was observed between the other four investigated SNPs and NSH. The comparison of heterozygous genotype and homozygous variant genotype frequency vs wild-type genotype frequency was statistically significant (C/T vs C/C analysis: chi-square = 7.47, *p*=0.006, OR = 2.54, 95% CI = 1.34–4.81; T/T vs C/C analysis: chi-square = 6.16, *p*=0.013, OR = 5.53, 95% CI = 1.46–20.87).

Regarding the allele frequency of the investigated SNPs, we noticed that the variant T allele of the MSX1 rs8670 SNP was more frequent in the NSH group (*p*=0.0002, OR = 2.65, 95% CI = 1.6–4.39). Based on our findings, we considered that the presence of the variant allele or variant genotype of the MSX1 rs8670 SNP increased the risk of hypodontia in the studied population.

Table 3 shows the genotype distributions and allele frequencies for the same five SNPs; comparison was made between the frontal agenesis NSH subjects and their sex- and age-matched controls. The two SNPs of the MSX1 gene seem to have an important role in hypodontia. Our results revealed that the variant genotype of the MSX1 rs12532 SNP (A/G + G/G) is more frequent in frontal agenesis (*p*=0.049, OR = 0.37), and this may be explained with the statistically significant difference of variant allele presence in these cases (*p*=0.048, OR = 0.49). When heterozygous genotype and homozygous variant genotype frequency was compared separately with wild-type genotype frequency, differences were not significant (A/G vs A/A analysis: chi-square = 2.99, *p*=0.08, OR = 0.39, 95% CI = 0.15–1.006; G/g vs A/A analysis: chi-square = 1.45, *p*=0.22, OR = 0.34, 95% CI = 0.08–1.37).

We also noticed that the variant genotype of the MSX1 rs8670 SNP (C/T + T/T) was more frequent in cases with frontal agenesis (*p*=0.0005, OR = 6.6, 95% CI = 2.29–18.96). The comparison of heterozygous genotype and homozygous variant genotype frequency vs wild-type genotype frequency was statistically significant (C/T vs C/C analysis: chi-square = 8.08, *p*=0.0045, OR = 5.1, 95% CI = 1.73–15.01; T/T vs C/C analysis: chi-square = 5.41, *p*=0.02, OR = 19.58, 95% CI = 1.02–372.66—no control subject had homozygous variant genotype). Based on our findings, we considered that the presence of the variant genotype of the MSX1 rs8670 SNP increased the risk of agenesis in the frontal region.

The risk of appearance of frontal agenesis is 6.15 times higher in the case of presence of the variant T allele of the MSX1 rs8670 SNP (*p*=0.0001, OR = 6.15, 95% CI = 2.38–15.89).

For the PAX9 rs61754301 SNP, all frontal agenesis NSH subjects and controls had wild-type genotype frequency, which made our results noninterpretable from the statistical point of view.

In the case of the AXIN2 rs2240308 SNP, a higher frequency of the variant genotype (G/A + A/A) was observed with a trend towards statistical significance. The presence of the variant A allele of AXIN2 rs2240308 was a risk factor for frontal agenesis (*p*=0.044, OR = 1.96, 95% CI = 1.06–3.62). Moreover, when heterozygous genotype and homozygous variant genotype frequency was compared separately with wild-type genotype frequency, we observed that the presence of the variant genotype of AXIN2 rs2240308 was not associated with the risk of developing frontal agenesis (G/A vs G/G analysis: chi-square = 1.71, *p*=0.18, OR = 2.34, 95% CI = 0.8–6.87; A/A vs G/G analysis: chi-square = 3.52, *p*=0.06, OR = 4.28, 95% CI = 1.13–16.18).

Moreover, we investigated the genotype distribution and allele frequency of studied SNPs in MSX1, PAX9, and AXIN2 in controls and lateral agenesis (Table 4). Although neither the distribution of wild-type, homozygous, and heterozygous genotypes nor the allele distributions showed no statistically significant differences, we observed a trend towards statistical significance in the case of PAX9 rs4904155, and the variant G allele of the mentioned SNP was more frequently found in cases with lateral agenesis (*p*=0.085, OR = 1.81).

## 4. Discussion

Our findings showed a greater prevalence in female patients, which is in agreement with most of the studies conducted on Caucasian subjects [7, 13, 14]. 42 cases (43.29%) had only frontal teeth missing, and 47 (48.45%) had only lateral teeth missing. 8.26% of agenesis cases were mixed, with incisor and bicuspid agenesis. The most prevalent missing teeth in frontal agenesis subjects were lateral incisors and in lateral agenesis cases were the second bicuspid; missing of these two types of teeth was found as the most frequent agenesis in most of the studies [3, 14, 15].

From the 42 subjects with incisor agenesis, 21 had upper bilateral lateral incisor agenesis; meanwhile in the lateral agenesis group, 19 subjects had bilateral lower second bicuspid agenesis. These results allow us to declare that, in our studied NSH group, bilateral agenesis occurs more often, and we found frontal agenesis more often in the upper arch, while lateral agenesis appears more frequently in the lower arch. The difference between upper and lower agenesis was not significant, and these findings were different from some of available data. Some of the studies we followed found considerably more frequent upper agenesis [15, 16], and others found a maxillary : mandibular agenesis overall ratio of 1.45 : 1 in orthodontic patients [17, 18].

Most often reported genes associated with nonsyndromic tooth agenesis are PAX9, MSX1, EDA, and AXIN2 [19]. SNPs of these genes have an impact on agenesis patterns although in notably different ways. The MSX1 gene (former homeobox 7 (HOX7)) is a nonclustered homeobox protein located on the chromosome 4p16.3-p16.1, and it was mentioned as the gene whose deletion results in complete failure of incisor development [20]. PAX9 is a member of the paired box (PAX) family of transcription factors, is located on the chromosome 14q12-q13, and was shown to be associated with all forms of the disease including autosomal dominant, nonsyndromic and familial oligodontia [20]. AXIN2 or axis inhibitor protein 2 is a gene located on the chromosome 17q23-q24 and is involved in sporadic forms of common incisor agenesis [21].

We studied two SNPs for MSX1 (rs12532 and rs8670), two for PAX9 (rs61754301 and rs4904155), and one for the AXIN2 gene (rs2240308). We analysed these polymorphisms separately for frontal and lateral NSH, which we consider to be the real value of this study. Some reviews were focused on the SNPs we also have studied, but none of these treated separately the differently located agenesis.

Regarding our findings, when we compared the genotypes in NSH and control groups, the only SNP which was statistically highly significant by its frequency was MSX1 rs8670. A significant association was observed between the heterozygous genotype of the MSX1 rs8670 SNP and its 2.54 times higher risk of developing NSH; meanwhile, the risk of developing hypodontia is 5.53 times for the homozygous variant genotype.

In the frontal agenesis group, the two mentioned polymorphisms of the MSX1 gene showed that the variant genotype is the most frequent one (highly significant in MSX1 rs8670, *p*=0.0005); meanwhile, variant-type alleles were identified in both groups as predominant alleles (highly significant in MSX1 rs8670, *p*=0.0001). The presence of heterozygous genotype increases the risk of frontal agenesis 5.1 times (*p*=0.0045), and this risk is 19.58 times higher when the homozygous variant genotype exists (*p*=0.02). The two above-mentioned SNPs showed no statistically significant differences in the lateral agenesis group. This SNP was not reported in congenital second premolar and upper lateral incisor agenesis so far [22].

As far as PAX9 rs61754301 and rs4904155 SNPs are considered, our findings were not significant as it seems that these polymorphisms have no influence on the expression of hypodontia in our subjects. Some of the studies reported MSX1 and PAX9 mutations in molar and bicuspid agenesis, and the PAX9 gene was positively associated with hypodontia susceptibility [23].

Studying the AXIN2 rs2240308 SNP, the only significant result was the variant allele dominancy in the frontal agenesis control group. A higher frequency of the variant genotype of AXIN2 rs2240308 was observed in patients with frontal agenesis than in controls (*p*=0.082) but without statistically significant association. In frontal agenesis, for this SNP, we found variant alleles more frequently in the NSH group. Additional common variants in AXIN2 have also been associated with increased susceptibility to hypodontia in Eastern Europeans [24].

## 5. Limitations

In our study, the diagnosis of NSH was declared based only on clinical and radiological examination, without a full phenotypic investigation. NSH subjects and controls were primarily Romanian Europeans, and this might mean that our findings cannot be generalized to all the population. Another limitation of our study is that a large number of SNPs could not be further tested and that many of the genes we preselected to study could not be completely interrogated.

## 6. Conclusions

In our study, NSH was more frequent in female patients, and bilateral upper lateral incisor agenesis and bilateral lower second bicuspid agenesis were the most frequent types of NSH we have found. In conclusion, the results of the present study showed that the variant genotype and variant T allele of the MSX1 rs8670 SNP increased the risk of hypodontia in the studied population, and it might be considered a risk factor for frontal agenesis The presence of the variant A allele of AXIN2 rs2240308 is associated with frontal agenesis but not with lateral agenesis.

## Figures and Tables

**Table 1:** Characteristics of the studied groups.

	NSH		Control		Frontal agenesis		Control		Lateral agenesis		Control	
	*n*	Mean age	*n*	Mean age	*n*	Mean age	*n*	Mean age	*n*	Mean age	*n*	Mean age
Male	27	15.51 ± 4.54	27	15.40 ± 3.82	13	19.07	13	18.78	12	15.61	12	15.40
Female	70	17.98 ± 7.84	70	17.80 ± 8.00	29	±7.62	29	±8.25	35	±6.00	35	±5.49
Total	97		97		42		42		47		47	

**Table 2:** Genotype and allele frequencies of MSX1, PAX9, and AXIN2 polymorphisms in the local population.

Polymorphism	Agenesis: all cases (*N* = 97), *n*	Controls (*N* = 99), *n*	Statistical analysis for genotypes	Statistical analysis for alleles
MSX1 rs12532 A/G				
A/A	50	45	A/G + G/G vs A/A	G vs A
A/G	37	42	Chi-square = 0.5	Chi-square = 0.53
G/G	10	12	*p*=0.477	*p*=0.462
A	137	132	OR = 0.78	OR = 0.83
G	57	66		

MSX1 rs8670 C/T				
C/C	49	74	C/T + T/T vs C/C	T vs C
C/T	37	22	Chi-square = 11.24	Chi-square = 14.09
T/T	11	3	*p*=0.0008^*∗*^	*p*=0.0002^*∗*^
C	135	170	OR = 2.9	OR = 2.65
T	59	28		

PAX9 rs61754301 C/T				
C/C	97	99	C/T + T/T vs C/C	T vs C
C/T	0	0		
T/T	0	0	NA	NA
C	194	198		
T	0	0		

PAX9 rs4904155 C/G				
C/C	40	50	C/G + G/G vs C/C	G vs C
C/G	43	34	Chi-square = 1.34	Chi-square = 0.61
G/G	14	15	*p*=0.246	*p*=0.432
C	123	134	OR = 1.45	OR = 1.2
G	71	64		

AXIN2 rs2240308 G/A				
G/G	18	24	G/A + A/A vs G/G	A vs G
G/A	49	55	Chi-square = 0.63	Chi-square = 2.32
A/A	30	20	*p*=0.426	*p*=0.127
G	85	103	OR = 1.4	OR = 1.39
A	109	95		

*p* ≤ 0.001, highly significant; 0.001 ≤ *p* ≤ 0.05, significant; *p* ≥ 0.05, not significant; NA, not available.

**Table 3:** Genotype and allele frequencies of MSX1, PAX9, and AXIN2 polymorphisms in frontal agenesis.

Polymorphism	Frontal agenesis (*N* = 42), *n*	Matched controls (*N* = 42), *n*	Statistical analysis for genotypes	Statistical analysis for alleles
MSX1 rs12532				
A/G	25	15	A/G + G/G vs A/A	G vs A
A/A	13	20	Chi-square = 3.86	Chi-square = 3.89
A/G	4	7	*p*=0.049^*∗*^	*p*=0.048^*∗*^
G/G	63	50	OR = 0.37	OR = 0.49
A	21	34		
G				

MSX1 rs8670 C/T				
C/C	20	36	C/T + T/T vs C/C	T vs C
C/T	17	6	Chi-square = 12.05	Chi-square = 15.08
T/T	5	0	*p*=0.0005^*∗*^	*p*=0.0001^*∗*^
C	57	78	OR = 6.6	OR = 6.15
T	27	6		

PAX9 rs61754301				
C/T	42	42	C/T + T/T vs C/C	T vs C
C/C	0	0		
C/T	0	0	NA	NA
T/T	84	84		
C	0	0		
T				

PAX9 rs4904155				
C/G	18	22	C/G + G/G vs C/C	G vs C
C/C	20	14	Chi-square = 0.42	Chi-square = 0.02
C/G	4	6	*p*=0.512	*p*=0.868
G/G	56	58	OR = 1.46	OR = 1.11
C	28	26		
G				

AXIN2 rs2240308				
G/A	7	15	G/A + A/A vs G/G	A vs G
G/G	23	21	Chi-square = 3.01	Chi-square = 4.03
G/A	12	6	*p*=0.082	*p*=0.044^*∗*^
A/A	37	51	OR = 2.77	OR = 1.96
G	47	33		
A				

*p* ≤ 0.001, highly significant; 0.001 ≤ *p* ≤ 0.05, significant; *p* ≥ 0.05, not significant; NA, not available.

**Table 4:** Genotype and allele frequencies of MSX1, PAX9, and AXIN2 polymorphisms in lateral agenesis.

Polymorphism	Lateral agenesis (*N* = 47), *n*	Matched controls (*N* = 47), *n*	Statistical analysis for genotypes	Statistical analysis for alleles
MSX1 rs12532 A/G				
A/A	21	20	A/G + G/G vs A/A	G vs A
A/G	21	19	Chi-square = 0.04	Chi-square = 0.21
G/G	5	8	*p*=0.835	*p*=0.646
A	63	59	OR = 0.91	OR = 0.82
G	31	35		

MSX1 rs8670 C/T				
C/C	25	33	C/T + T/T vs C/C	T vs C
C/T	19	13	Chi-square = 2.2	Chi-square = 2.57
T/T	3	1	*p*=0.137	*p*=0.108
C	69	79	OR = 2.07	OR = 1.9
T	25	15		

PAX9 rs61754301 C/T				
C/C	47	47	C/T + T/T vs C/C	T vs C
C/T	0	0		
T/T	0	0	NA	NA
C	94	94		
T	0	0		

PAX9 rs4904155 C/G				
C/C	18	26	C/G + G/G vs C/C	G vs C
C/G	22	18	Chi-square = 2.09	Chi-square = 2.96
G/G	7	3	*p*=0.147	*p*=0.085
C	58	70	OR = 1.99	OR = 1.81
G	36	24		

AXIN2 rs2240308 G/A				
G/G	9	12	G/A + A/A vs G/G	A vs G
G/A	22	24	Chi-square = 0.24	Chi-square = 1.07
A/A	16	11	*p*=0.62	*p*=0.306
G	40	48	OR = 1.44	OR = 1.4
A	54	46		

*p* ≤ 0.001, highly significant; 0.001 ≤ *p* ≤ 0.05, significant; *p* ≥ 0.05, not significant.

## Data Availability

The data used to support the findings of this study are available from the corresponding author upon request.

## References

[1] Rakhshan V., Rakhshan H. (2016). Meta-analysis and systematic review of the number of non-syndromic congenitally missing permanent teeth per affected individual and its influencing factors. *The European Journal of Orthodontics*.

[2] Rakhshan V. (2015). Congenitally missing teeth (hypodontia): a review of the literature concerning the etiology, prevalence, risk factors, patterns and treatment. *Dental Research Journal*.

[3] Laganà G., Venza N., Borzabadi-Farahani A., Fabi F., Danesi C., Cozza P. (2017). Dental anomalies: prevalence and associations between them in a large sample of non-orthodontic subjects, a cross-sectional study. *BMC Oral Health*.

[4] Hagiwara Y., Uehara T., Narita T., Tsutsumi H., Nakabayashi S., Araki M. (2016). Prevalence and distribution of anomalies of permanent dentition in 9584 Japanese high school students. *Odontology*.

[5] Azza Husam A.-A., Antoun J. S., Thomson W. M., Merriman T. R., Farella M. (2017). Hypodontia: an update on its etiology, classification, and clinical management. *BioMed Research International*.

[6] Yin W., Bian Z. (2015). The gene network underlying hypodontia. *Journal of Dental Research*.

[7] Larmour C. J., Mossey P. A., Thind B. S., Forgie A. H., Stirrups D. R. (2005). Hypodontia—a retrospective review of prevalence and etiology. *Quintessence International*.

[8] Shahid M., Joshi S., Alqhtani N. R. (2017). Single nucleotide polymorphism (SNPs) in the genes associated with tooth agenesis. *European Journal of Experimental Biology*.

[9] Bonczek O., Bielik P., Krejčí P. (2018). Next generation sequencing reveals a novel nonsense mutation in MSX1 gene related to oligodontia. *PloS One*.

[10] Abu-Siniyeh A. A., Khabour O. F., Owais A. I. (2018). The role of PAX9 promoter gene polymorphisms in causing hypodontia: a study in the Jordanian population. *The Application of Clinical Genetics*.

[11] Zhang T., Zhao X., Hou F. (2019). A novel PAX9 mutation found in a Chinese patient with hypodontia via whole exome sequencing. *Oral Diseases*.

[12] Yu M., Wong S. W., Han D., Cai T. (2019). Genetic analysis: Wnt and other pathways in nonsyndromic tooth agenesis. *Oral Diseases*.

[13] Behr M., Proff P., Leitzmann M. (2011). Survey of congenitally missing teeth in orthodontic patients in Eastern Bavaria. *European Journal of Orthodontics*.

[14] Laganà G., Lombardi C. C., Franchi L., Cozza P. (2011). Tooth agenesis: dentoskeletal characteristics in subjects with orthodontic treatment need. *European Journal of Paediatric Dentistry*.

[15] Tunc E. Ş., Bayrak S., Koyuturk A. E. (2011). Dental development in children with mild-to-moderate hypodontia. *American Journal of Orthodontics and Dentofacial Orthopedics*.

[16] Laganà G., Venza N., Lione R., Chiaramonte C., Danesi C., Cozza P. (2018). Associations between tooth agenesis and displaced maxillary canines: a cross-sectional radiographic study. *Progress in Orthodontics*.

[17] Fekonja A. (2005). Hypodontia in orthodontically treated children. *European Journal of Orthodontics*.

[18] Gomes R. R., Fonseca J., Paula L. M., Faber J., Acevedo A. C. (2010). Prevalence of hypodontia in orthodontic patients in Brazil. *European Journal of Orthodontics*.

[19] Mastouri M. H., De Coster P., Zaghabani A. (2016). Characterization of a novel mutation in PAX9 gene in a family with non-syndromic dental agenesis. *Archives of Oral Biology*.

[20] Kapadia H., Mues G., D’Souza R. (2007). Genes affecting tooth morphogenesis. *Orthodontics and Craniofacial Research*.

[21] Wong S., Liu H., Bai B. (2014). Novel missense mutations in the AXIN2 gene associated with nonsyndromic oligodontia. *Archives of Oral Biology*.

[22] De Muynck S., Schollen E., Matthijs G., Verdonck A., Devriendt K., Carels C. (2004). A novel MSX1 mutation in hypodontia. *American Journal of Medical Genetics*.

[23] Zhang W., Qu H. C., Zhang Y. (2014). PAX-9polymorphism may be a risk factor for hypodontia: a meta-analysis. *Genetics and Molecular Research*.

[24] Mostowska A., Biedziak B., Jagodzinski P. P. (2006). Axis inhibition protein 2 (AXIN2) polymorphisms may be a risk factor for selective tooth agenesis. *Journal of Human Genetics*.

